# Successful treatment for retroperitoneal cavernous hemangioma adjacent to the renal hilum via the laparoscopic approach: a case report

**DOI:** 10.1186/1752-1947-8-73

**Published:** 2014-02-26

**Authors:** Tsukasa Igawa, Shin-ichi Watanabe, Toru Onita, Hideki Sakai

**Affiliations:** 1 Department of Nephro-urology, Nagasaki University Graduate School of Biomedical Sciences, 1-7-1 Sakamoto, Nagasaki 852-8501, Japan

**Keywords:** Cavernous hemangioma, Retroperitoneal tumor, Laparoscopic surgery

## Abstract

**Introduction:**

Cavernous hemangiomas are common benign tumors of the skin or liver but can also rarely originate from the retroperitoneal space, especially adjacent to the renal hilum. Qualitative characterization of these retroperitoneal tumors using available imaging modalities is relatively difficult.

**Case presentation:**

A 40-year-old Japanese woman was incidentally noted to have a round homogenous tumor adjacent to the left renal hilum on computed tomography. The preoperative diagnosis was paraganglioma according to hormonal and clinical findings. The tumor was successfully resected via a laparoscopic approach, and histopathological examination of the tumor revealed cavernous hemangioma.

**Conclusions:**

Cavernous hemangioma is a rare but relatively benign disease when considering the different types of retroperitoneal tumors. We were able to effectively treat the retroperitoneal cavernous hemangioma via laparoscopy.

## Introduction

Retroperitoneal neoplasms are relatively rare, but widespread use of sophisticated imaging studies has resulted in an increase in the number of cases of retroperitoneal neoplasms that are detected. Among retroperitoneal tumors, cavernous hemangioma (CH) is even rarer [1,2]. Since the pathological features of retroperitoneal tumor are often difficult to diagnose on various imaging modalities, the operative approach is often the only practical treatment strategy. The methods of operative treatment may be one of the important discussion points due to various anatomical features of tumors, that is, tumor size and location. The present report describes a case of a patient with a cavernous hemangioma adjacent to the left renal hilum. This tumor was successfully resected using a laparoscopic approach.

## Case presentation

**Figure 1:**
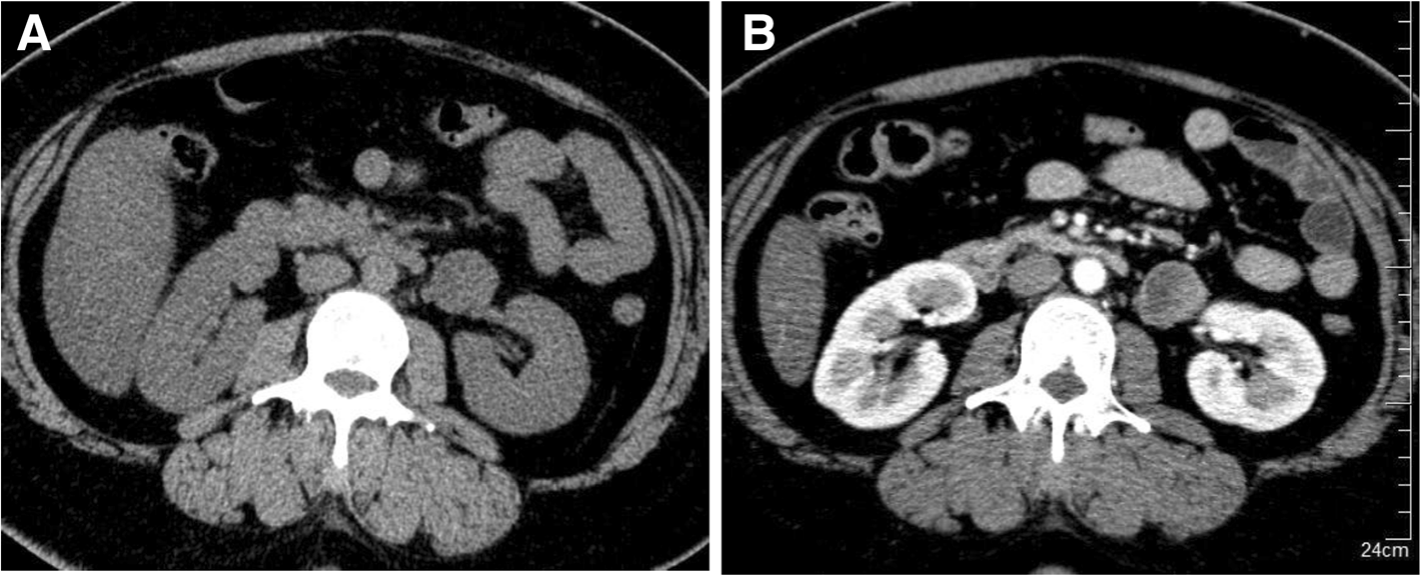
**Abdominal computed tomography findings.** Plain computed tomography showed a homogenous round tumor of 4×3×2cm in size **(A)** and weak enhancement in the peripheral area of the tumor **(B)**.

**Figure 2:**
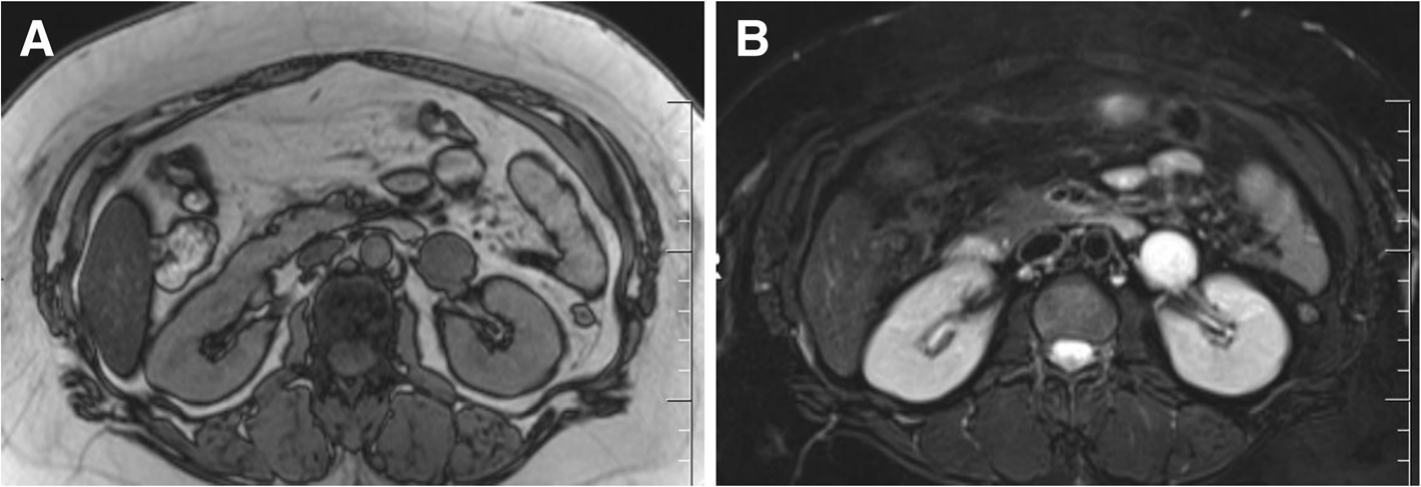
**Magnetic resonance imaging findings. (A)** T1-weighted images showed a tumor with low signal intensity and **(B)** very high signal intensity on T2-weighted images.

A 40-year-old Japanese woman was referred to our department due to a retroperitoneal tumor. Originally, she planned to undergo surgical repair of an umbilical hernia, but pre-operative evaluation by computed tomography (CT) revealed a round-shaped solid mass, 4×3×3cm in size, located in the retroperitoneum just below the left renal vein. The tumor was gradually and weakly enhanced beginning in its peripheral area (Figure 1). Magnetic resonance imaging (MRI) showed a tumor with low signal intensity on T1-weighted images and very high signal intensity on T2-weighted images (Figure 2). Our patient had been undergoing antihypertensive treatment for three years and complained of a mild headache at the time of consultation; therefore, we first suspected paraganglioma. A hormonal study and ^123^I-metaiodobenzylguanidine (MIBG) scintigraphy were performed, showing an elevated plasma norepinephrine level (702pg/ml; normal range, 100 to 450pg/ml) and a normal plasma epinephrine (32pg/ml; normal range, <100pg/ml) and dopamine (10pg/ml; normal range, <20pg/ml) level. A 24-hour urine collection demonstrated a slight increase in normetanephrine level (0.38pg/ml; normal range, 0.1 to 0.28pg/ml). However, ^123^I-metaiodobenzylguanidine (MIBG) scintigraphy did not show high uptake in the tumor area. Based on these results, we could not completely rule out paraganglioma, and our patient was started on doxazosin (2mg daily, with stepwise dose-escalation to a maximum dose of 14mg daily).

**Figure 3:**
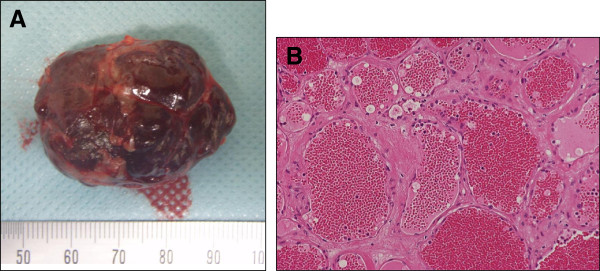
**Macroscopic findings and histopathology of the resected specimens. (A)** The tumor was capsulized and was dark brick in color. **(B)** Microscopic examination of the specimen showed dilated and congested vascular spaces lined by a single layer of endothelial cells, hematoxylin and eosin (HE) staining × 40.

Next, surgical removal of the tumor was performed. Our patient was placed in a modified lateral decubitus position (approximately 60 degrees) and underwent surgery via the transperitoneal approach using three ports and standard laparoscopic devices under general anesthesia. No severe adhesion was observed during the surgical procedure, and the tumor was radically resected in 1.5 hours, with an estimated blood loss of 5ml. The cut surface of the tumor appeared dark brick-colored and had a homogenous macroscopic appearance (Figure 3A). Histopathological examination revealed poorly circumscribed and irregularly dilated blood vessels filled with red blood cells, and the blood vessels were lined by a flat endothelium (Figure 3B). A diagnosis of cavernous hemangioma was made. Postoperative blood pressure and plasma catecholamine levels were similar to that observed before the operation. Our patient has not experienced any recurrence over the two-year follow-up period.

## Discussion

CH most commonly affects the skin, mucosa and liver but can rarely occur in the retroperitoneal space. Several reports of CH originating from retroperitoneal organs (for example, adrenal gland, kidney and pancreas) are available, but primary retroperitoneal CH is even rarer [1,2]. CH is essentially a benign neoplasm that produces clinical symptoms only when the tumor compresses an adjacent organ or tissue. Most reported cases of retroperitoneal CH involve symptoms of abdominal pain when the tumor grows to a large size (for example, approximately 10cm in diameter). However, most cases of CH are asymptomatic and are only incidentally detected on routine imaging, as occurred in our present case. Based on the tumor location, MRI findings and abnormal catecholamine studies in our present case, we could not rule out paraganglioma and elected to utilize surgical resection as the treatment strategy. In regard to imaging findings, our present case showed relatively slow and weak enhancement on CT imaging, and enhancement was patchy in the peripheral area of the tumor compared with paraganglioma. This observation may be a diagnostic feature that is specific to retroperitoneal CH and could be used to differentiate retroperitoneal CH from paraganglioma. However, the CT findings of retroperitoneal CH have not been extensively characterized [3,4]. Further, CT findings of CH may differ depending on the organ of origin. For example, typical CH of the pancreas shows strong contrast enhancement when compared with that of normal pancreas tissue [5]. He *et al.* described a well-encapsulated retroperitoneal CH without enhancement of the inner component [2]. Similar to CT, MRI findings of retroperitoneal CH have not been well established. In general, CH shows low signal intensity on T1-weighted images and relatively high signal intensity on T2-weighted images. However, the degree of signal intensity might vary depending on the specific tumor component. Despite the availability of various imaging modalities, the differential diagnosis of retroperitoneal tumors adjacent to renal hilum still includes neurogenic tumors (for example, neuroganglioma, schwannoma), and lymphangiogenic tumors, (for example, lymphoma, IgG4-related disease), and other rare soft tissue tumors (for example, sarcomas). Thus, accurate pre-treatment diagnosis of retroperitoneal tumors remains difficult [6].

Usually, watchful waiting is a common treatment strategy for asymptomatic and small CHs. In fact, hepatic CH is treated only when the tumor is large or produces symptoms. However, Forbes reported spontaneous rupture of adrenal hemangioma, which manifested as severe pain and hypotension [7]. Further, Takeda *et al.* reported rapidly growing CH with continuous abdominal pain [8]. In addition, as in our present case, a diagnosis of paraganglioma or other malignant tumors is often difficult to exclude. These observations indicate that routine follow-up and adequate therapeutic intervention are sometimes required even for small retroperitoneal CHs. As for the therapeutic intervention, surgical resection could be the only and most effective treatment for retroperitoneal tumors at the moment. Other treatment modalities, for example, kinds of molecular targeted therapy, chemotherapy or radiation therapy, are tentatively applied for a small portion of cases.

In our present case, the laparoscopic approach was utilized for tumor resection. Since the tumor was located close to large vessels, (that is, left renal hilar vessels and abdominal aorta), meticulous manipulation was required and surgical resection might be challenging for this type of tumor. However, a laparoscopic procedure under magnified view can be a reasonable and safe approach when conducted by an experienced surgeon. In addition, postoperative recovery is much faster after laparoscopic resection when compared with recovery after open laparotomy. However, unexpected tumor adhesion or invasion can sometimes be appreciated during the laparoscopic approach, and these findings often necessitate conversion to an open approach. Recent studies suggest that laparoscopic resection is a safe and feasible operative approach for hemangioma of the adrenal gland or for paraganglioma [9,10]. Thus, the laparoscopic approach might represent a new standard operative procedure for the surgical management of retroperitoneal tumor. More precise preoperative diagnosis of retroperitoneal tumors would be of benefit to facilitate selection of the optimal treatment strategy.

## Conclusions

Our report describes a rare case of retroperitoneal CH adjacent to the renal hilum that was difficult to distinguish from paraganglioma based on the clinical findings. CH should be considered within the differential diagnosis of retroperitoneal tumors, and a patchy CT enhancement pattern in the peripheral area of the tumor might be indicative of a diagnosis of CH. The laparoscopic route represents a feasible and safe surgical approach for these tumors.

## Consent

Written informed consent was obtained from the patient for publication of this case report and accompanying images. A copy of the written consent is available for review by the Editor-in Chief of this journal.

## Abbreviations

CH: Cavernous hemangioma; CT: Computed tomography; MRI: Magnetic resonance imaging.

## Competing interests

The authors declare that they have no competing interests.

## Authors’ contributions

SW and TO cared for the patient. HS helped to write the discussion. All authors read and approved the final manuscript.

## Authors’ information

Shin-ichi Watanabe, Toru Onita and Hideki Sakai are co-authors.
